# Success rates of trial of labor after cesarean delivery: the impact of prior vaginal deliveries on outcomes

**DOI:** 10.1007/s00404-025-08248-4

**Published:** 2026-01-28

**Authors:** Yaara Bashan, Elior Shalev Maman, Ron Rosenberg, Yael Yekel, Amir Weintraub, Nir Ram Duvdevani, Yael Pasternak

**Affiliations:** 1 The Adelson School of Medicine, Ariel University, HaRav Yisha’ayahu Meshorer 22, Petach Tikva, Israel; 2 Laniado Medical Center, Netanya, Israel

**Keywords:** Vaginal birth after cesarean delivery (VBAC), Cesarean delivery (CD), Uterine rupture, TOLAC (trial of labor after cesarean), Dystocia

## Abstract

**Objectives:**

To estimate the success rates and risks of vaginal birth after cesarean delivery (VBAC) based on the number of prior successful VBACs.

**Methods:**

A retrospective cohort study of women with one cesarean section in the past who attempted vaginal delivery between 2013 and 2022, using data from our Medical Center registry. Outcomes were compared based on the number of prior successful VBACs.

**Results:**

Among 2912 deliveries meeting the eligibility criteria, the success rate of VBAC increased with the number of prior VBACs: 73.2% for those with no prior VBAC, rising to 92.3%, 94.7%, 94.0%, and 97.0% for individuals with 1, 2, 3, 4, and 5 or more prior VBACs, respectively. The history of at least one prior VBAC was associated with a 5.17-fold higher likelihood of achieving VBAC success. However, no significant differences in success rates were observed between groups with higher numbers of prior VBACs (≥ 2) compared to individuals with only one prior VBAC. In addition, the duration of hospitalization for both mother and neonate was longer in cases with no prior VBAC history. There was also a higher risk of requiring blood transfusion in the group without a prior history of VBAC.

**Conclusions:**

Women with prior successful VBAC have a high likelihood of achieving another successful VBAC. After two prior VBACs, the success rate remains stable. In addition, women with one or more previous VBACs experience a reduced risk of blood transfusion and shorter hospitalization durations for both the mother and newborn.

## What does this study add to the clinical work

**Table:** 

Women with a prior successful VBAC have a much higher chance of another successful VBAC, with no added benefit from multiple prior VBACs. A repeat trial of labor after cesarean is generally safe and may reduce hospital stay and the need for blood transfusions for both mother and baby.	

## Introduction

After a cesarean delivery (CD), two options are available for subsequent births: elective repeat cesarean delivery or a trial of labor after cesarean (TOLAC). Each option has its own advantages and disadvantages.

Elective recurrent CD is known to be associated with a higher incidence of complications, such as placenta previa and placenta accreta [1, 2]. However, repeat cesarean delivery reduces the overall risk of complications of TOLAC, mainly by avoiding the risks associated with TOLAC failure, such as uterine rupture [3–5]. While the risk of uterine rupture is low [5–8], it is associated with increased maternal and fetal morbidity and mortality [9, 10].

Several factors predict the success of TOLAC [11, 12], with one of the most significant being the history of a successful VBAC. Previous VBAC success is associated with higher likelihoods of subsequent VBAC success, reduced risk of uterine rupture, and lower rates of other complications [13–16].

Some studies have explored whether there is a difference in the success rate of TOLAC among women who have had more than one VBAC following a cesarean delivery. The findings indicate that after the first VBAC, additional successful VBACs do not increase the success rate of subsequent TOLAC attempts [15, 16]. It was not shown yet, though, that the higher number of VBACs in the past increases the success rate in the current TOLAC. In this study, we aim to further investigate the influence of the number of VBACs in the patient’s history on the success rate of the current TOLAC.

## Materials and methods

This retrospective study was conducted at a single center between 2013 and 2022. The study collected data on women who had undergone a single cesarean section and chose to attempt a TOLAC.The study included only women with a singleton pregnancy, whose first cesarean section was performed at our hospital, and for whom the indication for the first cesarean was known.

Excluded from the study were women with multiple pregnancies, fetuses with congenital anomalies or chromosomal abnormalities, women who attempted vaginal birth after two cesarean sections, and women whose first cesarean was not performed at our hospital or for whom the reason and for the first cesarean was unknown as well as its timing.

The study group consisted of women who had not previously experienced a successful vaginal birth after cesarean, making this their first VBAC attempt. The control group included women who had already undergone one or more successful vaginal births after a cesarean, with some having one, two, three, four, or five or more vaginal deliveries following a cesarean section.

Demographic data that we collected included: maternal age, Gestational age, Parity, Reason for the first cesarean section, Months between CS to TOLAC and the newborn’s weight and dystocia as reason for the first CD. The maternal and neonatal outcomes that were recorded included length of maternal hospitalization after delivery, the need for blood transfusion, uterine rupture, and bladder rupture, Newborn Apgar score, Neonatal admission to the neonatal intensive care unit and the newborn’s length of hospitalization.

The primary outcome was the success of vaginal birth after cesarean (VBAC) in women with no prior history of VBAC compared to those with a previous successful vaginal delivery following a cesarean. In addition, we examined whether the number of previous successful vaginal deliveries influenced the outcome.

As secondary outcomes, we assessed whether there were more maternal complications, as previously mentioned, along with increased hospitalization days. We also evaluated whether there were more neonatal complications, such as an Apgar score below 7 at 5 min, admission to neonatal intensive care, or an increase in the newborn’s hospitalization days after delivery.

In addition, we investigated whether a history of dystocia, which was the reason for the initial cesarean delivery, was also a contributing factor to failure during TOLAC, ultimately leading to a repeat cesarean section.

Due to the retrospective nature of the research and the extraction of anonymized data from a computerized database, individual informed consent was waived. The study received Helsinki approval, number 0009-23-LND.

Data were collected from the hospital's computer system and analyzed using SPSS software. To compare the groups, we conducted logistic regression analysis to calculate odds ratios (ORs) with 95% confidence intervals (95% CI). Univariate logistic regression was initially used to assess the relationship between each independent variable and the outcome variable. For multivariate logistic regression, multiple independent variables were included in the model to account for potential confounders. In cases where expected frequencies were low or sample sizes were small, Fisher’s exact test was applied to assess associations between categorical variables.

## Results

**Fig. 1:**
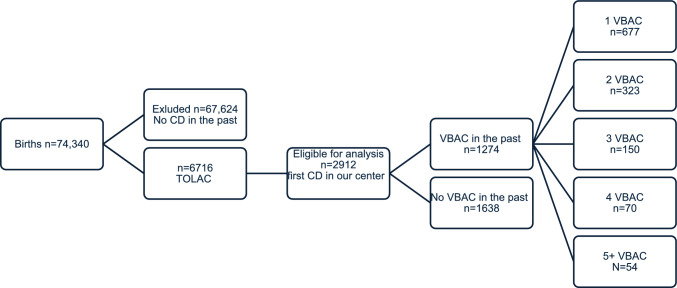
Patient selection for the trial

During the study period, a total of 74,340 births took place at our medical center. Of these, 6716 women attempted a TOLAC with a singleton pregnancy. Among them, 2912 had their previous cesarean delivery performed in our center, allowing access to both the documented indication for the cesarean and the time interval since the procedure (Fig. 1).

Table 1 presents demographic variables of the two groups. The first group included women who had one or more previous VBACs after their CD. The second group included women who had not had a VBAC after their cesarean section, and this was their first TOLAC. The variables presented include the woman’s age, Parity, gestational week, number of months between the CD and the TOALC, newborn weight, and whether the reason for the first cesarean section was dystocia. All variables, except dystocia, demonstrated statistically significant *p* values.

**Table 1:** Demographic characteristics of the two group, Group 1 includes women who had a previous VBAC after their CD

	VBAC in the past (*n* = 1274)	No VBAC in the past (*n* = 1638)	*p* value
Age	32.73 ± 4.7	30.98 ± 5.16	< 0.001
Parity	3.93 ± 2.22	1.906 ± 1.678	< 0.001
WP	39.94 ± 1.64	39.75 ± 1.76	0.002
Months between CS to TOALC	80.09 ± 33.13	34.74 ± 21.02	< 0.001
Newborn weight	3.35 ± 0.49	3.28 ± 0.49	< 0.001
Dystocia in the past	149 (11.69%)	227 (13.85%)	0.686

Values are presented as a mean standard deviation or number (rates%)

Group 2 women who did not have a VBAC after their CD, and this is their first TOLAC

Table 2 presents the outcomes for each group. The success rate of TOLAC was significantly higher in the first group-women who had a prior VBAC, with a success rate 5.17 times greater compared to the second group. Since some of the demographic characteristics were significantly different between the 2 groups, logistic regression was required to clarify whether a difference in success rate is still noticed. After adjusting for all demographic variables through logistic regression, the success rate remained significantly higher in the first group, with an odds ratio of 4.85.

**Table 2:** Maternal and neonatal outcome comparing the two groups, Group 1 includes women who had a previous VBAC after their CD. Group 2 women who did not have a VBAC after their CD, and this is their first TOLAC

Maternal and neonatal outcome	VBAC in the past (*n* = 1274)	No VBAC in the past (*n* = 1638)	OR (CI 95%)
Successful delivery	1190 (93.4)	1200 (73.3)	5.17 (4.04–6.62)
Bladder rupture	0 (0.0)	3 (0.2)	0.262*
Uterine rupture	3 (0.2)	9 (0.5)	0.55 (0.22–1.34)
Blood Transfusion	23 (1.8)	58 (3.5)	0.50 (0.31–0.82)
APGAR < 7	8 (0.6)	19 (1.2)	0.54 (0.24–1.22)
Neonatal ICU	45 (3.5)	75 (4.6)	0.76 (0.52–1.11)
Hospitalization day mother	3.8 ± 3	4.2 ± 2.5	**< 0.001
Hospitalization day neonate	4.4 ± 3.1	4.8 ± 5	0.009**
Recurrent dystocia	2 (0.15%)	31 (1.89%)	**< 0.001

Values are presented as a mean standard deviation or number (rates%)

^*^Fisher's exact test

^**^p value

Rates of bladder and uterine rupture showed no significant differences between the groups. However, blood transfusions were significantly more common in the second group, composed of women without a prior VBAC.

Neonatal outcomes, including low APGAR score at 5 min and NICU hospitalization rates, showed no significant differences between the groups. On the other hand, the length of hospitalization for both mothers and neonates was significantly shorter in the first group compared to the second.

Recurrence of dystocia as a reason for failure in a TOLAC was significantly more frequent in the group without a prior successful VBAC.

**Fig. 2:**
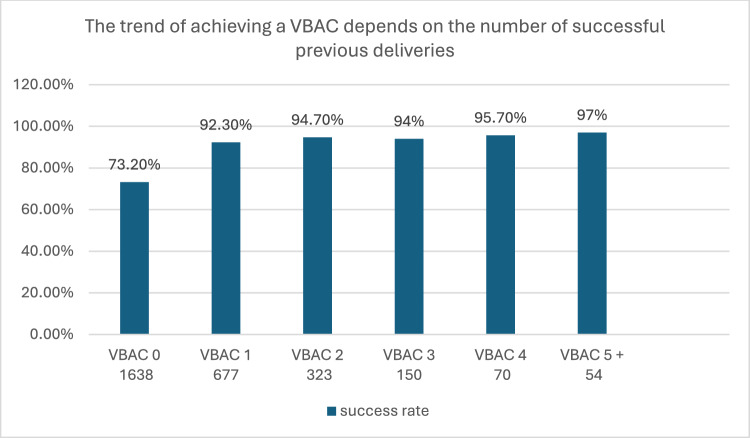
VBAC success rates increase with the number of prior successful VBAC deliveries. Percentages above bars indicate success rates; numbers below categories denote sample sizes

Figure 2 shows the success rate of VBAC by the number of prior VBACs, reaching 73.2%, 92.3%, 94.7%, 94%, 95.7% and 97% for women with 0, 1, 2, 3 4 and 5 and up prior VBACs, respectively. The success rate increased significantly between the no VBAC and one VBAC, but did not change significantly with added numbers of VBACS.

Out of 1274 women who had previously undergone VBAC, 84 women experienced unsuccessful attempts at vaginal delivery and were transferred to cesarean section. The most common reason for cesarean section as a standalone factor was fetal distress, accounting for 31 cases (36.9%). This was followed by dystocia, with 15 cases (17.85%), and placental abruption, with 5 cases (5.95%). Other reasons 33 cases (39.28%) varied and included conditions, such as pre-eclampsia, malpresentation that was presented at labor time, the woman's request to halt the process, and more.

In the second group Out of 1638 women who had not previously undergone VBAC, 438 underwent cesarean section. The reasons for the surgeries were varied. The most common standalone reason was fetal distress, with 135 cases (30.82%). The second most common reason was dystocia, with 93 cases (21.23%), followed by placental abruption, with 12 cases (2.7%). The remaining cases, accounting for 198 instances (45.2%), were due to various other reasons, as mentioned earlier.

Table 3 presents the reasons for the unsuccessful VBAC in the 2 groups. For all the reasons for TOLAC failure, no statistically significant differences were observed.

**Table 3:** Reasons for unsuccessful VBAC in the two groups

Reasons for failure at the TOLAC	VBAC in the past (*n* = 84)	No VBAC in the past (*n* = 438)
Fetal distress	31 (36.9%)	135 (30.82%)
dystocia	15 (17.85%)	93 (21.23%)
Placenta abruption	5 (5.95%)	12 (2.7%)
Other *	33 (39.28%)	198 (45.2%)

^*^Pre-eclampsia, malpresentation, the woman's request to halt the process, and more

## Discussion

Women with a history of one successful VBAC have a 5.3 times higher likelihood of achieving another successful VBAC compared to women who have never experienced a VBAC. Due to demographic differences between the groups, we performed logistic regression. The results show that even after performing multivariable analysis for potential confounders, the likelihood of a successful VBAC is 4.85 times higher if there was a previous VBAC. The number of deliveries following the cesarean section, after one VBAC has already occurred, does not significantly impact the likelihood of a successful VBAC. These results are consistent with findings reported in other studies [15, 16]. These results can be explained by the reasons for the initial cesarean delivery. As can be seen from the results of our data for the first CD, there was no statistical difference in the rates of dystocia between the groups. However, the recurrence rate of dystocia was significant, with more cases of dystocia observed in the group without prior VBAC. This indicates that our ability to predict dystocia in the first delivery is not precise, as is well-known in obstetrics. Therefore, what provides us with more information about CPD (Cephalopelvic Disproportion) is the outcome of a subsequent delivery, whether successful or a failure that results in a CD. When a woman is diagnosed with CPD or dystocia, these are often relative diagnoses rather than absolute ones, as we lack definitive tests to confirm their validity [17, 18] As we have observed, the recurrence rate of this diagnosis as a cause for failed TOALC is low, ranging from 0.15% in women with a prior VBAC to 1.89% in those without a previous VBAC. Given the significant difference between these groups, a successful VBAC strongly suggests that the woman has an adequate pelvic structure, reinforcing the likelihood that she can deliver vaginally again.

In addition, women who previously had a successful VBAC required fewer blood transfusions and had shorter hospital stays for both mother and baby, findings that were statistically significant. These findings align with the results reported in other studies [4, 14]

There were no significant differences in the rates of bladder injury, low Apgar scores, or neonatal intensive care unit (NICU) admissions between the groups. These findings are consistent with results reported in previous studies [4, 19].

Our analysis demonstrated a trend toward a lower incidence of uterine rupture in women with a history of one or more prior VBACs compared to those attempting their first VBAC. While the rate of uterine rupture was lower in the group with prior VBACs (0.2%) compared to the group without (0.5%), This difference did not reach statistical significance, which may be attributed to the low incidence of uterine rupture and the potential need for a larger sample size to detect a significant effect. This trend aligns with prior research that reported a significantly lower risk of uterine rupture among women with prior VBACs (0.4–0.5%) compared to those attempting their first VBAC (0.9%, *p* < 0.01) [15, 20, 21].

The leading reason for performing cesarean sections in women without prior VBAC was suspected fetal distress. This aligns with the fact that fetal distress is often the first clinical sign of uterine rupture [22–24]. As a result, we tend to adopt a more cautious approach and opt for a cesarean section more readily.

The strengths of this study include its relatively large sample size, based on a single center, allowing for consistent data collection and reliable tracking of the indications for the first cesarean delivery, which were all performed at our institution. This enabled us to compare the reasons for the first and second cesarean sections and demonstrate a statistically significant difference in dystocia as a recurrent cause for cesarean delivery in the group with no previous successful VBAC. Nevertheless, the likelihood of this cause recurring remains low. Consequently, this study group is smaller, but unlike other studies in the field, we were able to highlight this due to the availability of detailed data.

These findings are consistent with previously reported results in the literature [13, 16, 19, 20, 24] and contribute additional data to further reinforce existing knowledge.

However, this study has several limitations. Its retrospective design precluded the collection of data on certain variables, such as BMI and placenta accreta spectrum disorders. In addition, as the study was conducted at a single center, the generalizability of the findings may be limited.

Future research in this area could benefit from multicenter studies with a broader focus on more diverse and generalized populations to enhance the external validity of the findings.

In addition, in the era of artificial intelligence, developing machine learning models tailored to predict TOLAC success on a patient-specific basis could significantly advance clinical decision-making. By integrating individual patient history and other relevant factors, such models have the potential to improve prediction accuracy and personalize counseling for patients considering TOLAC.

## Conclusion

This data highlights the benefits of pursuing repeat TOLAC as a safe option for patients with a history of VBAC even if the reason for the first CD was dystocia or CPD, due to the low likelihood of recurrence of this diagnosis. Specifically, it demonstrates reductions in hospitalization durations for both mother and baby, as well as decreased need for blood transfusions. These advantages, in turn, contribute to a reduction in healthcare system costs. Furthermore, the high success rates of vaginal delivery underscore the favorable benefit–risk ratio, which should be clearly communicated to this patient subpopulation during counseling for a trial of labor. These findings are particularly significant in populations with a cultural or personal preference for larger families, where multiple deliveries are common. For such patients, TOLAC presents a much more favorable option in terms of reducing long-term maternal morbidity associated with repeated cesarean sections.

## Data Availability

No datasets were generated or analysed during the current study.
